# Experimental analysis on cyanide removal of gold tailings under medium-temperature roasting

**DOI:** 10.1038/s41598-023-28842-3

**Published:** 2023-03-07

**Authors:** Long Hai, Xianglong Fang, Xin Zhao, Bo Xu, Tongjun Cheng

**Affiliations:** School of Mechanics and Engineering, Liaoning Technical University, Fuxin, 123000 Liaoning China

**Keywords:** Materials science, Engineering

## Abstract

The cyanide content of gold tailings exceeds the standard seriously due to the cyanide extraction process. In order to improve the resource utilization efficiency of gold tailings, a medium-temperature roasting experiment was carried out on the stock tailings of Paishanlou gold mine after washing and pressing filtration treatment. The thermal decomposition rule of cyanide in gold tailings was analyzed, and the effects of different roasting temperatures and roasting durations on cyanide removal efficiency were compared. The results show that when the roasting temperature reaches 150 °C, the weak cyanide compound and free cyanide in the tailings begin to decompose. When the calcination temperature reached 300 °C, the complex cyanide compound began to decompose. When the roasting temperature reaches the initial temperature of cyanide decomposition, the cyanide removal efficiency can be improved by prolonging the roasting time. After roasting at 250–300 °C for 30–40 min, the total cyanide content in the toxic leachate decreased from 3.27 to 0.01 mg/L, which met the water quality standard of III class in China. The research results provide a low-cost and efficient way for cyanide treatment, which is of great significance for promoting the resource utilization of gold tailings and other cyanide-containing wastes.

## Introduction

As a typical representative of solid waste, gold tailings are still less than 40% in comprehensive utilization rate because of the excessive cyanide content. In order to reduce the mining cost of mining enterprises and avoid the pollution of the mining environment caused by a large number of cyanide-containing tailings, a high-efficiency, low-cost, and no secondary pollution cyanide removal process is developed to realize the harmless treatment of cyanide-containing tailings. The primary prerequisite for the utilization of tailings resources.

At present, the methods of removal cyanide from tailings mainly includes physical methods, chemical methods, hydrolysis methods and incineration methods^1–5^. The physical method mainly adopts the solid–liquid separation and washing method, which can reduce the cyanide content in the toxic leaching solution of tailings to below 5 mg/L^6–8^. The method has simple process, no secondary pollution, high treatment efficiency, relatively low treatment cost, and a wide range of applications. The solid–liquid separation and washing method is determined by the "Technical Specification for the Control of Cyanide Residue Pollution in the Gold Industry" to be the preferred cyanide removal process of the cyanide-containing tailings used for backfilling materials. However, no chemical reaction occurs during the washing process, some of the water-insoluble complex cyanide compound in the tailings cannot be removed by the washing method. At the same time, due to the technological limitations of the washing method, it is inevitable that some cyanide will remain in the tailings slag. This method leads to low cyanide removal efficiency, and is only suitable for the basic process of cyanide removal from tailings.

The chemical method uses chemical reagents to oxidize cyanide in tailings into relatively weak and easily hydrolyzed cyanic acid (HCNO), which is then removed by further oxidation and hydrolysis. According to the choice of oxidant, it can be divided into: chlorine oxidation method, INCO method, ozone oxidation method, and hydrogen peroxide oxidation method. The chlorine oxidation method has the advantages of low cost, simple procedure and high degree of automation, and has a good removal effect on weak cyanide compounds in tailings, but the removal effect of complex cyanide compounds, especially ferricyanide compounds is not obvious, and it is easy to cause The treated tailings contain some residual chlorine and incompletely reacted cyanogen chloride, which will cause corrosion to the cyanide removal equipment and cause secondary pollution^9–11^. INCO method can not only effectively remove weak cyanide compounds, but also has good removal effect on complex cyanide compounds and ferricyanide compounds. However, some thiocyanate compounds cannot be removed, and the metal ions in the complexed cyanide compounds are prone to form carbonate, hydroxide and ferricyanide precipitation, which are attached to the surface of tailings particles. INCO method is more suitable for treating cyanide-containing wastewater with low thiocyanate content, when it is used for cyanide-containing tailings slag removal treatment, ferricyanide and thiocyanate cannot be completely removed, so that cyanide in tailings cannot be completely removed,and it is difficult to continue to reduce the content of cyanide^12,13^. The ozone oxidation method can oxidatively decompose other cyanides except ferricyan compounds (including but not limited to thiocyanate compounds and heavy metal complexes) into nitrogen and bicarbonate radicals, and has a faster reaction rate. In the whole process of ozone removal of cyanide, no secondary pollutants are introduced, and the treatment process is simple, but the cost of cyanide removal is greatly increased due to the expensive ozone generating equipment, high energy consumption, easy failure, and difficult maintenance^14,15^. Ozone oxidation is usually used as an auxiliary process to carry out advanced treatment of low concentration cyanide containing tailings. The reaction speed of cyanide removal by hydrogen peroxide method is extremely fast, and the cyanide content can be reduced to the emission requirements in a short time. However, this process has no obvious effect on the treatment of ferricyanide and thiocyanate compounds, and is prone to produce cyanate precipitation, which adheres to the surface of the tailings and reduces the cyanide removal efficiency^16,17^. At the same time, the preparation process of hydrogen peroxide is complex, expensive, and there are great potential safety hazards, resulting in a relatively narrow scope of application of the cyanogen peroxide oxidation method.As a result, it is only suitable for deep cyanide removal treatment of cyanide-containing waste liquid with low content of thiocyanate and ferricyanide.

The "three wastes" collaborative purification method is to use sulfur-containing flue gas or oxidizing liquid to remove cyanide from cyanidation wastewater and washing liquid, recycle the wastewater and exhaust gas, and treat the waste residue to meet the standard, so as to achieve waste treatment with waste^18–20^. It essentially oxidizes cyanide through sulfur dioxide, but due to its poor oxidant properties, its ability to remove cyanide is much lower than that of Inco method. At present, the "three wastes" synergistic purification method for cyanide removal from tailings can effectively reduce the cyanide content in tailings to less than 30 mg/L. The cyanide removal ability is relatively poor, and it can be applied to high-concentration tailings which has not been treated by pressing-washing and removing cyanide.

The hydrolysis method does not require chemical agents, does not produce secondary pollution, moreover the equipment operation and maintenance process are simple. The main cost of cyanide removal comes from the purchase of high temperature and high pressure treatment equipment and the energy consumption of operation. It is widely used in the decyanation treatment of refractory cyanide-containing wastewater^21,22^.However, the high cost and high energy consumption of the current high temperature and high pressure treatment device limit the wide application of the hydrolysis method. Compared with other chemical methods for removing cyanide, the hydrolysis method is more suitable for the removal of cyanide from tailings in mining enterprises with excess energy output from nearby power plants and relatively low energy consumption costs, especially when the complexed cyanide in the tailings is used. Or when the content of thiocyanate is high, it is more advantageous to use the hydrolysis method to remove cyanide.

The incineration method is mainly based on the cement kiln co-processing method. The use of gold tailings to replace some of the cement raw materials for cement production has the advantages of complete cyanide removal and simple process. However, in order to ensure that the quality of cement is not affected, only a small amount of tailings can be mixed, making it difficult to improve the efficiency of tailings treatment^23,24^.

At present, the cyanide removal process is limited by the high cost of chemicals, low processing efficiency and poor processing capacity, and cannot be widely used in the mine filling industry. In this paper, the existing high-temperature incineration process for removing cyanide was improved, and the muffle furnace was used to conduct a medium-temperature roasting experiment on the tailings in Liaoning Paishanlou Company after washing and filter-pressing. On the basis of maintaining low energy consumption, the roasting temperature and roasting time are selected to make the residual cyanide content in the tailings reach the standard, and an industrialized cyanide removal scheme is designed to provide a low-cost and high-efficiency new way for the removal of cyanide from cyanide-containing tailings and improve the tailings. Improving the resource utilization efficiency of tailings can produce huge economic, environmental and social benefits.

## Experiment

The cyanide content detection of tailings in this article was assisted by Paishanlou company which has the qualified of cyanide content detection. The detection standard is that the total cyanide content in the tailings toxic leaching solution is less than 0.05 mg/L, while the national standard GB/T 14848-2017 "Quality of Groundwater" The standard for the detection of cyanide content in Class III water requires that the content of easily released cyanide is less than 0.05 mg/L (hereinafter, the total cyanide content in the tailings toxic leaching solution is referred to as the total cyanide content of tailings). Total cyanide includes easy-to-release cyanide and difficult-to-release cyanide, and Paishanlou's testing standards are stricter than those in the specification.

### Experiment material

**Figure 1:**
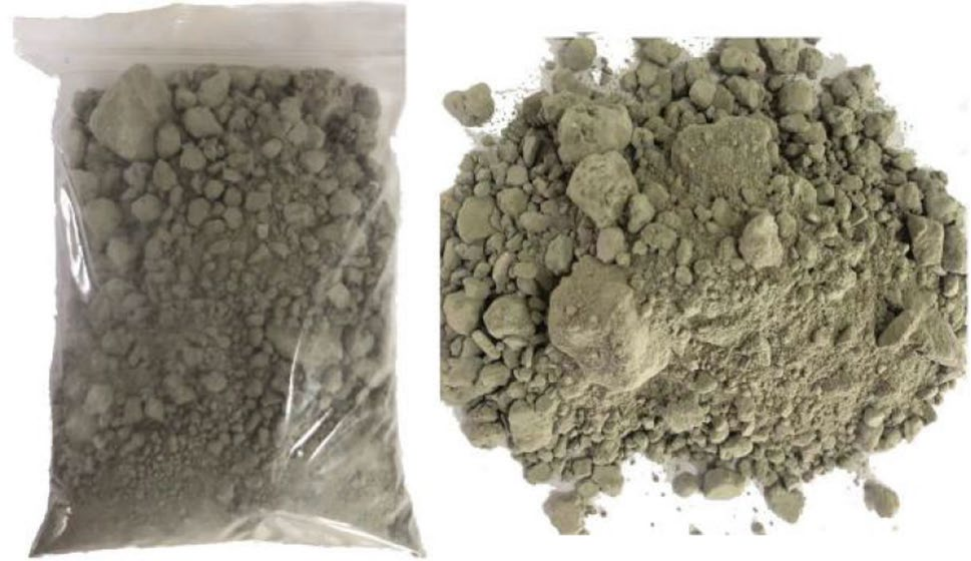
Tailings packing and sample morphology diagram.

The experiment material is the tailings with low cyanide content in Paishanlou Company tailings pond which has been washed and filtered. The total cyanide content in the tailings was 3.27 mg/L, the initial water content was 17.8%, and the liquid index was 0.13, which was in a hard plastic state. Paishanlou Company was entrusted to assist in sampling, and the tailings were put into sealed bags to avoid spilling during sample transportation and pollute the surrounding environment. At the same time, it could also ensure that the moisture content and cyanide content of the samples were not disturbed by external factors. Figure 1 shows the tailings package and the morphology of the sample.

### Experiment method

Cyanide-containing tailings are roasted by using SX2-12-12A box-type resistance furnace (muffle furnace) with XMT (TDW) temperature control regulator. When the tailings temperature reaches above 150 °C, the decomposition rate of cyanide will increase significantly. Due to the limitation of energy consumption and cyanide removal efficiency, the heating temperature is controlled within 400 °C, and the roasting time is controlled within 40 min. The initial heating temperature was selected as 150 °C, and was gradually increased to 350 °C with 50 °C as a gradient. A sample was taken every 10 min and sent to Paishanlou Company to detect the cyanide content.

The specific test steps are as follows:

1. Turn on the temperature controller, preheat the muffle furnace, and discharge the excess water inside the muffle furnace. The airtight door should be closed during the preheating process to avoid heat loss and to discharge the moisture attached to the airtight door. In order to prevent the furnace wall from cracking during the process of expelling moisture, the preheating temperature should be set to 75 °C first, and then increase the preheating temperature to 105 °C when there is no obvious moisture evaporation in the furnace.
2. The quartering method was used for sampling, and 1200 g of samples were weighed by an electronic balance and divided into 4 parts on average, each of which was 300 g (100 g for the moisture content test and 100 g for the cyanide content test. Due to the moisture content of the tailings The sum of the loss on ignition is about 20%, the quality of the sample will decrease by about 20% after roasting, and the remaining 240 g can meet the minimum requirements of the detection test.), naturally piled into the crucible, smoothed the surface and numbered, the stacking thickness is about 25 mm.
3. Increase the temperature of the muffle furnace to 150 °C. When the temperature is stable, open the sealing door of the muffle furnace, put 4 samples into the muffle furnace with crucible tongs, close the sealing door of the muffle furnace, and start timing. When the roasting time reaches 10 min, 20 min, 30 min and 40 min respectively, open the sealing door, take out one of the four samples with crucible tongs, close the sealing door, and put the sample into a sealing bag after cooling then label it well.
4. After all the samples of the previous group are roasted and put into a sealed bag. Repeating the sample making process of step (2). The temperature of the muffle furnace was gradually increased to 200 °C, 250 °C, 300 °C, and 350 °C, and the roasting process of step (3) was repeated.

## Results and discussion

### Experiment results

**Figure 2:**
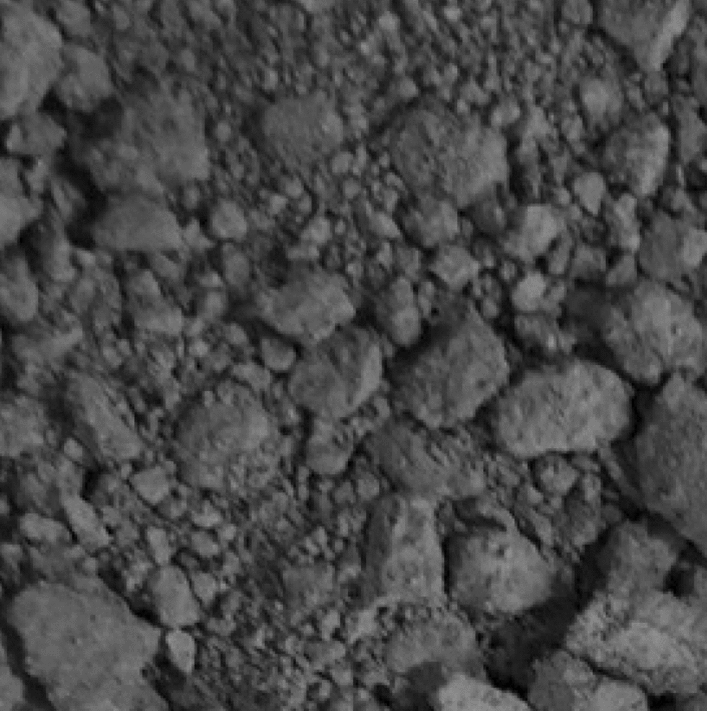
Tailings after roasting under 250 °C 10 min.

**Figure 3:**
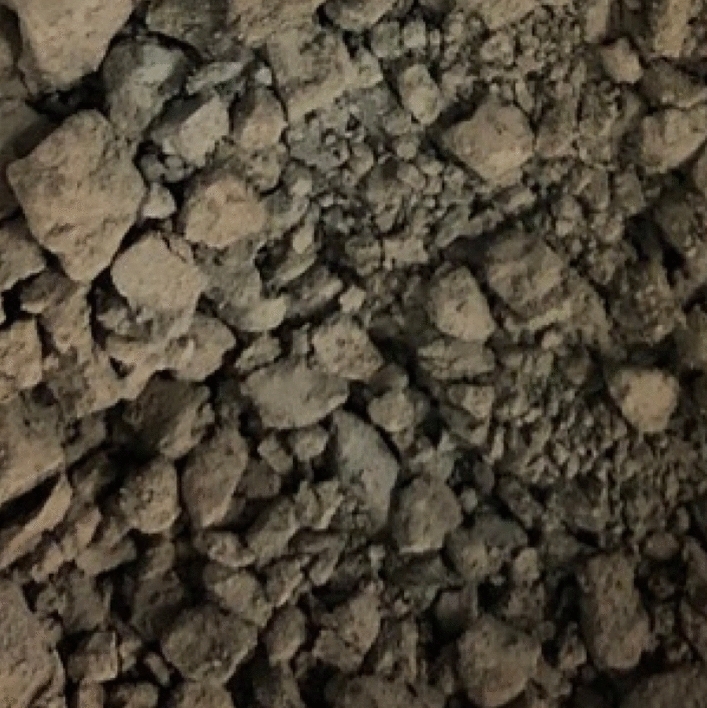
Tailings after roasting under 250 °C 20 min.

**Figure 4:**
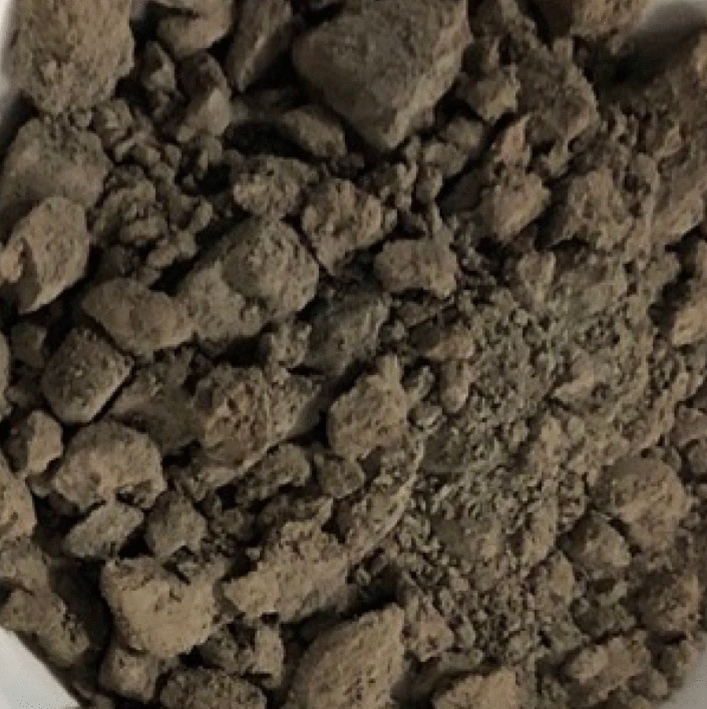
Tailings after roasting under 250 °C 30 min.

**Figure 5:**
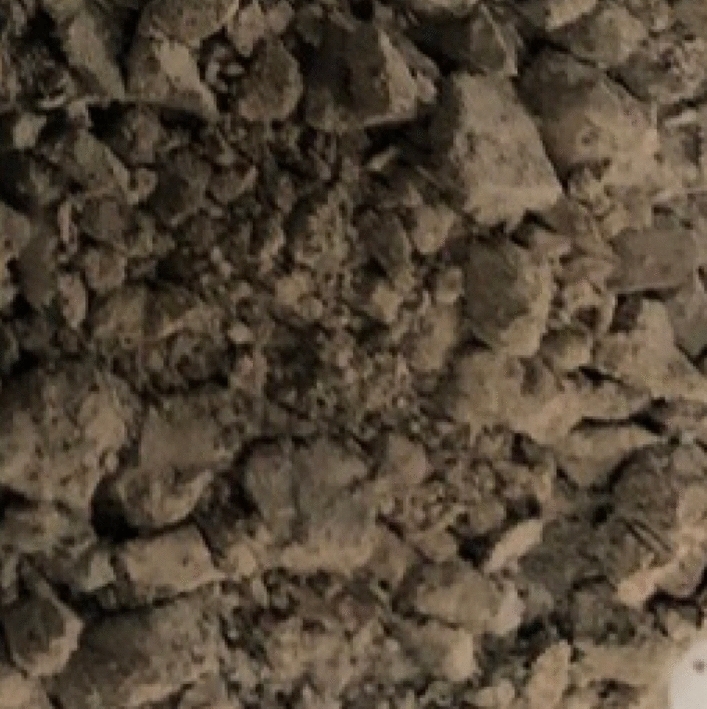
Tailings after roasting under 250 °C 40 min.

The experiment results are shown in Table 1. The morphology of tailings after roasting at 250 °C is shown in Figs. 2, 3, 4, and 5.

**Table 1:** experiment results of cyanide removal by roasting.

No	Temperature (°C)	Time (min)	Total cyanide content (mg/L)	Moisture content (%)	NO	Temperature (°C)	Time (min)	Total cyanide content (mg/L)	Moisture content (%)
1	150	10	0.62	7.12	11	250	30	0.01	0.18
2		20	0.40	2.4	12		40	0.01	0.12
3		30	0.21	0.43	13	300	10	0.23	2.52
4		40	0.16	0.29	14		20	0.18	2.21
5	200	10	0.65	5.51	15		30	0.01	0.11
6		20	0.14	2.33	16		40	Not detected	0.10
7		30	0.05	0.19	17	350	10	0.34	1.92
8		40	0.03	0.20	18		20	0.01	1.15
9	250	10	0.33	4.32	19		30	Not detected	0.14
10		20	0.19	3.31	20		40	Not detected	0.14

With the continuous roasting, the particle size of tailings does not change, and the color gradually changes from cyan to dark yellow. In addition, the smell of rotten eggs was obviously present during the cyanide removal test of tailings roasting, indicating that sulfur dioxide was generated from the decomposition of thiocyanide.

The minimum detectable total cyanide content is 0.01 mg/L. When the total cyanide content of the sample submitted for inspection is <0.01 mg/L, the cyanide content is marked as not detected. From the roasting test results, it can be concluded that the cyanide content and water content of the tailings samples are significantly reduced within the roasting time of 40 min. When the total cyanide content in the tailings is less than 0.05 mg/L, meet the related standards.

### Discussion

1. The effect of roasting temperature on the removal of cyanide.

**Figure 6:**
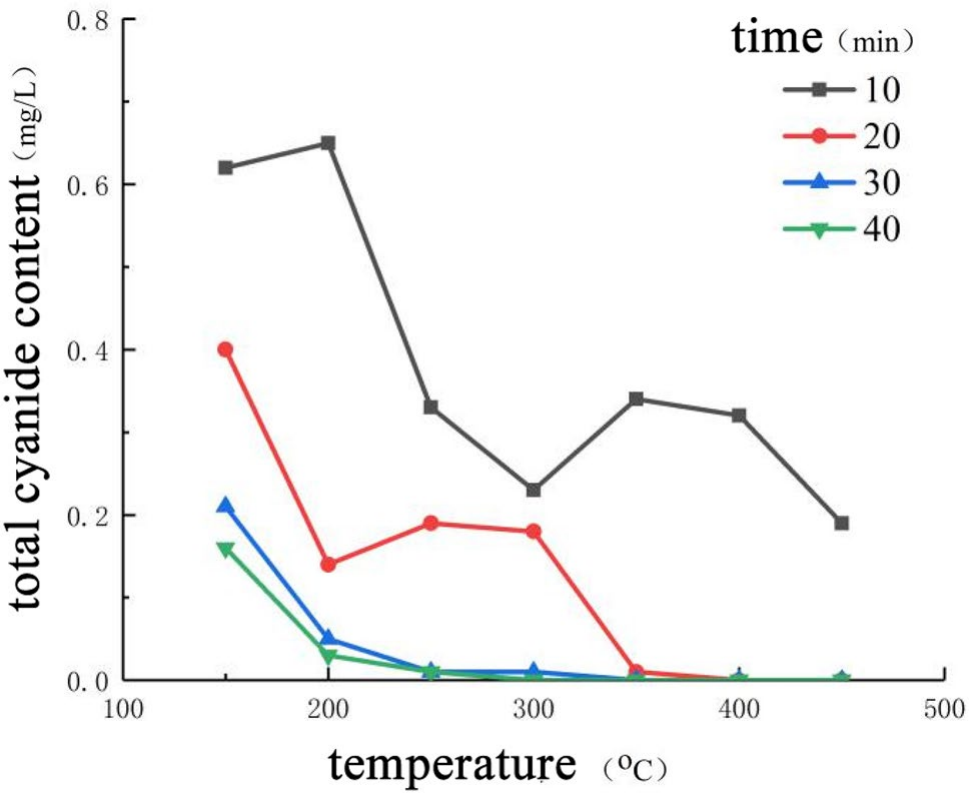
Curves of total cyanide content in tailings changing with roasting temperature.

Increasing the roasting temperature can significantly reduce the cyanide content in the tailings, as shown in Fig. 6 When the roasting time is less than 20 min, the roasting temperature increases, and the cyanide content in the tailings increases instead. This is because the roasting time is short and the heat in the furnace has not been transferred to the inside of the tailings heap, resulting in some tailings not being heated and the temperature difference is large. The tailings directly in contact with the muffle furnace or accumulated on the surface, the temperature rises rapidly, and the cyanide content decreases rapidly, while the tailings located in the middle of the accumulation body, the cyanide content decreases slowly.

When the roasting temperature is 150–200 °C, heating for 20 min can reduce the total cyanide content of tailings by 80.12–95.19%. Continuous heating for 40 min will continue to reduce the total cyanide content of tailings, but it cannot be reduced to below 0.05 mg/L. When the roasting temperature is higher than 300 °C, heating for 30–40 min can reduce the total cyanide content of tailings to below 0.05 mg/L.

(B) The effect of roasting time on the removal of cyanide.

**Figure 7:**
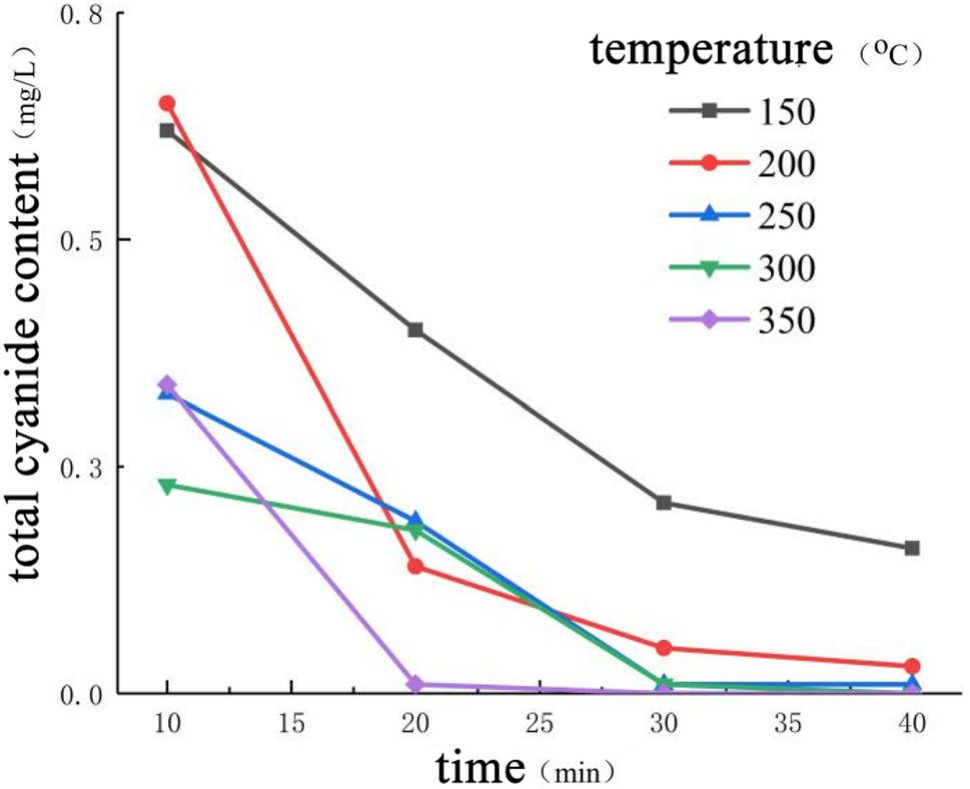
Relationship between cyanide content and roasting time in tailings.

The relationship between total cyanide content of tailings and roasting time is shown in Fig. 7 Extending the roasting time can effectively improve the removal rate of total cyanide in tailings. When the roasting time was within 10 min, the total cyanide content in the tailings decreased from the initial value of 3.27 mg/L to 0.34–0.62 mg/L, and the cyanide removal efficiency was 89.60%-81.03%, with the highest cyanide removal efficiency. However, due to insufficient roasting time, the tailings samples were heated unevenly, which caused large fluctuations in the cyanide content in the samples. Within 30–40 min of roasting, the total cyanide content of tailings decreased slightly, and the cyanide removal efficiency was extremely low. Simply prolonging the roasting time could not effectively reduce the total cyanide content of tailings. But at higher roasting temperature, continuous roasting has the strongest cyanide removal ability, which can effectively reduce the total cyanide content of tailings.

(C) Analysis on the principle of cyanide removal by roasting.

Cyanide in gold tailings mainly includes free cyanide, weak cyanide and strong cyanide. Free cyanide mainly refers to hydrogen cyanide (HCN) and cyanide ion (CN–). Hydrogen cyanide is easily soluble in water and produces cyanide ions, which can form hydrogen cyanide. The chemical properties of hydrogen cyanide molecules are relatively stable and can be slowly decomposed under the condition of light or heating. Weak cyanide compounds are ionic compounds formed by the reaction of hydrocyanic acid with metals such as zinc and iron in solution. These compounds are chemically unstable and can be decomposed into free cyanide compounds under natural conditions. Cyanide can also form complexes (coordination compounds) with copper, cobalt, gold and other elements in solution, that is, strong cyanide compounds. Compared with weak cyanide compounds, these heavy metal complexes are more stable and decompose very slowly.

Roasting can destroy the coordination bond inside the heavy metal complex cyanide compound and make it decompose; while the weak cyanide compound can be converted into free cyanide after roasting, mainly in the form of hydrogen cyanide molecules, and hydrogen cyanide molecules can be heated by continuous heating. Decomposed into non-toxic substances such as ammonia (NH_3_), acetic acid CH_3_COOH) and carbon dioxide (CO_2_).

The roasting and decomposition process of cyanide in cyanide-containing tailings at different temperatures can be divided into the following four stages.

① The first stage is the decomposition process of weak cyanide compounds and free cyanide in the tailings. During this process, the roasting temperature is lower than 200 °C and the roasting time is less than 20 min. The weak cyanide compounds and free cyanide in the tailings begin to decompose in large quantities, with Fastest cyanide removal rate. However, due to insufficient roasting time in this process, the decomposition of weak cyanide compounds and free cyanide compounds is not complete, and there are still many cyanide compounds attached to the tailings particles.
② In the second stage, the weak cyanide compounds and free cyanide have been largely decomposed. The roasting temperature reaches 200 °C, and the roasting time exceeds 20 min. The tailings temperature is relatively uniform. Although the cyanide removal efficiency has decreased, the complex cyanide compound has just begun to decompose gradually. At this time, the temperature in the furnace becomes the decisive factor affecting the decomposition of cyanide in the tailings. When the temperature is lower than 200 °C, the actual temperature of complex cyanide compound thermal decomposition is not reached, and the cyanide decomposition rate slows down. When the temperature is higher than 250 °C, it begins to enter the continuous decomposition stage of complex cyanide compound.
③ The third stage is the continuous decomposition stage of complex cyanide compound. At this time, only about 1% of complex cyanide compound with stable properties exists in the tailings. When the temperature in the furnace is higher than 250 °C and the roasting time is 30 min, the minimum requirement for the decomposition of complexed cyanide is met, and the decomposition rate of cyanide increases significantly with the increase of temperature, but it is still much lower than the previous two stages. The decomposition of cyanide can be further promoted by prolonging the calcination time or increasing the calcination temperature.
④ In the fourth stage, the tailings roasting temperature is 250–300 °C, and the roasting time is 30–40 min. The cyanide in the tailings enters the complete decomposition stage, Almost all cyanide is decomposed, and the total cyanide content in the tailings is less than 0.01 mg/L, it is difficult to detect and meet the relevant national standards.

(D) Roasting cyanide removal scheme.

Table 2 shows the cyanide removal efficiency of roasting scheme which cyanide content after tailings roasting that meet relevant standards.

**Table 2:** The cyanide removal efficiency of roasting scheme.

Time (min)	Temperature (^o^C)	Total cyanide content (%)	Cyanide removal efficiency (%)
40	200	0.03	99.08
30	250	0.01	99.69
40	250	0.01	99.69
30	300	0.01	99.69
40	300	Not detected	> 99.69
20	350	0.01	99.69
More than 30	More than 350	Not detected	> 99.69

By summarizing the research results, it can be seen that when the roasting temperature is 200 °C and the roasting time is 40 min, the cyanide content in the tailings is 0.03 mg/L, which is slightly lower than relevant standards; after 250–300 °C, 30–40 min roasting, most of the cyanide and thiocyanide in the tailings were removed, and the decomposition rate of cyanide in the tailings reached more than 99.69%. In order to avoid the interference of other factors and ensure that the total cyanide content of tailings reaches the standard, a scheme with better cyanide removal efficiency should be selected. When the roasting temperature is 250–300 °C and the roasting time is 30–40 min, the removal efficiency of cyanide is high, the energy consumption is low, and the total cyanide content of tailings can meet the relevant requirements, which is the best cyanide removal scheme.

During the roasting process, a small amount of cyanide will inevitably be volatilized into the air in the form of hydrogen cyanide and sulfur-containing flue gas. In industrial cyanide removal, a gas recovery system needs to be added to make it have a better absorption effect on cyanide gas and sulfide gas, and the waste gas can be reused in metallurgical processes or other industrial production. The basic research on the thermal decomposition of cyanide is still relatively weak. These analyses are conjectural conclusions based on comprehensive evaluation of the existing research results, and some verification experiments are still needed to draw valuable conclusions.

## Conclusions

1. Medium-temperature roasting can effectively reduce the cyanide content in tailings at a lower cost. It has the advantages of large processing capacity, high cyanide removal efficiency, strong ability, and wide application range, which can meet the needs of industrialized cyanide removal treatment of tailings.
2. The cyanide removal effect of tailings roasting is affected by roasting temperature and roasting time. The roasting temperature is used as the initial condition for the decomposition of cyanide. The free cyanide and weak cyanide compounds in the tailings have obvious decomposition effect when the temperature is higher than 150 °C, while the thermal decomposition of complex cyanide requires the roasting temperature to reach above 250 °C. When the roasting temperature reaches the initial condition of cyanide decomposition, the roasting time becomes the determining factor of the cyanide content of tailings. Full roasting for 30-40 min can completely remove cyanide in tailings, and the content is difficult to detect.
3. The tailings of Paishanlou Company after washed and filtered were roasted at 250–300 °C for 30–40 min, the total cyanide content in the toxic leachate was reduced from 3.27 mg/L to below 0.01 mg/L. It meets my country's Class III water quality standards.

## Data Availability

The datasets used and/or analysed during the current study available from the corresponding author on reasonable request.
